# Expression of Caspase-1 Gene Transcript Variant mRNA in Peripheral Blood Mononuclear Cells of Patients with Primary Gout in Different TCM Syndromes

**DOI:** 10.1155/2015/361607

**Published:** 2015-10-19

**Authors:** Wan-Tai Dang, Dan Xu, Wen-Guang Xie, Jing-Guo Zhou

**Affiliations:** ^1^School of Clinical Medicine, Chengdu University of Traditional Chinese Medicine, Chengdu 610075, China; ^2^Institute of Rheumatology and Immunology, Affiliated Hospital of North Sichuan Medical College, Nanchong 637000, China; ^3^Nephrology Department, Affiliated Hospital of North Sichuan Medical College, Nanchong 637000, China

## Abstract

A large number of studies have shown that cysteinyl aspartate specific protease-1 (CASP1) played an important role in the inflammatory response of primary gout, but the decreased expression of different CASP1 transcript variant could inhibit the activation of IL-1*β*. Our study mainly analyzed the expression level and function of CASP1 gene transcript variant mRNA in peripheral blood mononuclear cells of patients with gout in different TCM syndromes. The expression of CASP1 gene transcript variant and IL-1*β* mRNA in PBMCs were detected in patients with PG [acute phase (AP: 44 cases); nonacute phase (NAP: 52 cases)] and healthy controls (HC: 30 cases) by reverse transcription-polymerase chain reaction and/or real-time quantitative polymerase chain reaction. The expressions of plasma IL-1*β* in patients with PG and HC were detected by enzyme-linked immunosorbent assay. Dysregulated expression of the CASP1 gene and its transcript variant, plasma proinflammatory cytokines in all patients with primary gout in different TCM syndromes, correlation analysis showed that there was negative correlation between the expression of CASP1-gamma gene transcript variant mRNA and IL-1*β* protein in APPG group. The study suggested that CASP1 gene and its transcript variant may play a critical role in the inflammatory response of patients with PG in different phases and TCM syndromes.

## 1. Introduction

Gout is a clinical syndrome which is attributed to precipitation and deposition of monosodium urate (MSU) crystals on the tissue or organ caused by purine dysbolism and/or excretion reduction and continuous elevation of uric acid, and it belongs to metabolic rheumatism [1]. Gout is similar to Lijei or severe and migratory arthralgia in traditional Chinese medicine; its early elaboration is reported in* Ge Zhi Yu Lun* written by Zhu DanXi in which the pathogenesis of gout was regarded as phlegm, wind-heat, wind-wet and blood deficiency. Then, some doctors classified migratory Bi syndrome or painful Bi syndrome of Bi syndrome as gout. Recent researches have showed that inflammation and immunity are also involved in the pathogenesis of gout besides metabolism factors [2]. We know that the MSU released by aging and death cells in the body is endogenous danger-associated molecular patterns (DAMPs) caused by inflammation and apoptosis through innate immunity [3]. Cysteinyl aspartate specific protease-1 (CASP1) is also called IL-1*β* invertase, is involved mainly in regulation of inflammation, and plays an important role in the inflammatory response [4]. Luksch et al. [5] found that the decreased expression of different CASP1 gene transcript variant could reduce the activation of the IL-1*β*. Recent study showed that CASP1 played a key role in the course of gout [6, 7]. However, there was no study reporting the role of CASP1 gene transcript variant in different traditional Chinese medicine (TCM) syndromes of primary gout yet. In our study, the expression level of CASP1 gene transcript variant mRNA in peripheral blood mononuclear cells (PBMCs) of patients with primary gout (PG) in different TCM syndromes was measured by semiquantitative reverse transcription-polymerase chain reaction (RT-PCR) and/or real-time quantitative polymerase chain reaction (qRT-PCR); in the meantime, the interleukin 1*β* was measured to explore the role of CASP1 gene and its transcript variant in the pathogenesis of gout.

## 2. Methods

### 2.1. The Clinical Data

All the research objects conformed to the 1977 American Rheumatism Association (ACR) diagnostic criteria [8], excluding the objects which have secondary gout caused by diseases of kidney, cardiovascular and blood system, or drugs and so on and also excluding coinfection, autoimmune diseases, long-term use of hormone therapy or serious condition that may affect the efficacy and safety of our study [9, 10]. 96 male patients with PG who visited the Department of Rheumatology of the Affiliated Hospital of North Sichuan Medical College from December 2012 to October 2013 were admitted; among them 44 patients were in acute phase and 52 patients were in nonacute phase. The age of patients ranged from 23 to 79 years old, and the mean age was 40 ± 11 years with their disease course being 9 ± 3 years. 30 healthy people set as healthy controls (HC) were admitted from the Department of Physical Examination in the same hospital during the same period whose age ranged from 22 to 70 years, their mean age was 44 ± 7 years, and their laboratory indexes were normal, excluding the people who had diseases or family history of cardiovascular disease, diabetes, liver, gout, and so on [10]. The age between the two groups showed no statistical significance. The study has gained approval and agreement of the local ethics committee and all participants signed informed consent.

### 2.2. The TCM Syndromes

Based on the gout TCM syndrome differentiation in* Clinical Diagnosis and Treatment Terminology of Traditional Chinese Medicine-Syndrome Type Part* [11] and* Guideline of the Study on New Traditional Chinese Medicine* [12] and the TCM differentiation according to comprehensive analysis by the “*four examination methods,*” “*Eight Principle Pattern Identification,*” and “*Zang-fu pattern identification,*” the TCM syndromes differentiation of PG patients was divided into four types: obstruction of dampness and heat syndrome (ODHS), intermingled phlegm-stasis blood syndrome (IPSBS), Pi-deficiency induced dampness syndrome (PDIDS), and Qi-blood deficiency syndrome (QBDS) [13].

### 2.3. Main Reagents and Instruments

Human lymphocytes separation liquid (Batch number LTS10771) was product of Jingyang Tech Co., China; RNAiso Plus Reagent (Batch number A9701-1), PrimeScript RT reagent kit with gDNA eraser (Perfect qRT-PCR) kit (Batch number AK1801), SYBR Premix Ex Taq II (Batch number AK5004), and TaKaRa LA Taq kit (CKA4501A) were products of Takara BIO Inc., Japan; ELISA kits specific for human interleukin-1*β* (IL-1*β*) (Batch number 20131014) was product of Beijing 4A Biotech Co., Ltd., China. [7].

The 7900 real-time fluorescence quantitative PCR instrument was a product of ABI Company, USA. The hypothermic high-speed centrifugal machine 5417R was a product of Eppendorf Company, USA. The FlexCycler PCR instrument was a product of Analytik Jena AG, Germany. The FUSION-Fx5 lithography machine was a product of Oriental Science & Technology Development Co., Ltd., China [7].

### 2.4. Primer Design

**Figure 1:**
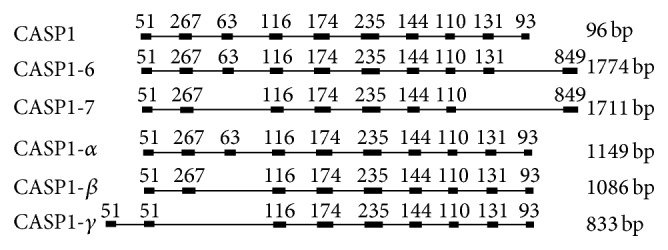
The exon of CASP1 gene and its transcript variant in human. Notes: rectangle: exon; straight line: intron; the number above the rectangle: length of exon; bp: fragment size.

Located in 11q23, the ID of CASP1 gene is 834, with ten exons, nine introns, seven transcript variants, and two functional domains (Figure 1). According to human *β*-actin and CASP1 gene and its transcript variant in GenBank, the primer was designed to be used in the measurement of RT-PCR or qRT-PCR and synthesized by polyacrylamide gel electrophoresis method in Genscript Biotechnology Co. Ltd., Designed primer sequences (Table 1).

### 2.5. Total RNA Extraction and cDNA Synthesis

**Figure 2:**
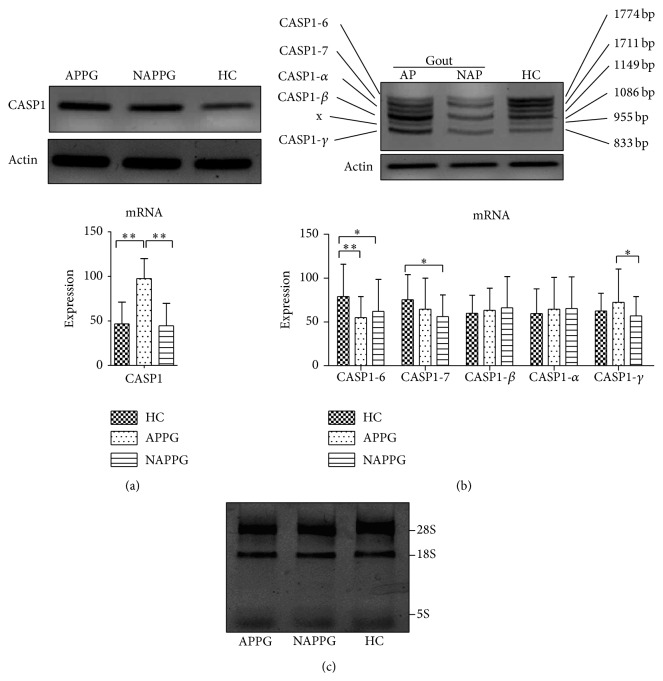
The mRNA expression of CASP1 gene and its transcript variant in PBMCs of patients with PG in different phases. Notes: HC: health control, AP: acute phase of primary gout, NAP: nonacute phase of primary gout; (a) CASP1 gene primers were amplified to one fragment (96 bp); (b) common primers of CASP1 gene transcript variants 6, 7, beta, alpha, and gamma were amplified to six fragments: 1774 bp was transcript variant 6, 1711 bp was transcript variant 7, 1086 bp was transcript variant beta, 1149 bp was transcript variant alpha, 833 bp was transcript variant gamma, and x was an unknown stripe. Delta and epsilon stripe were not observed in designed primers of our research, and we will carry on the design and experiment through different methods. (c) The RNA quality electropherogram of PBMCs. ^∗^ *P* < 0.05; ^∗∗^ *P* < 0.01.

**Figure 3:**
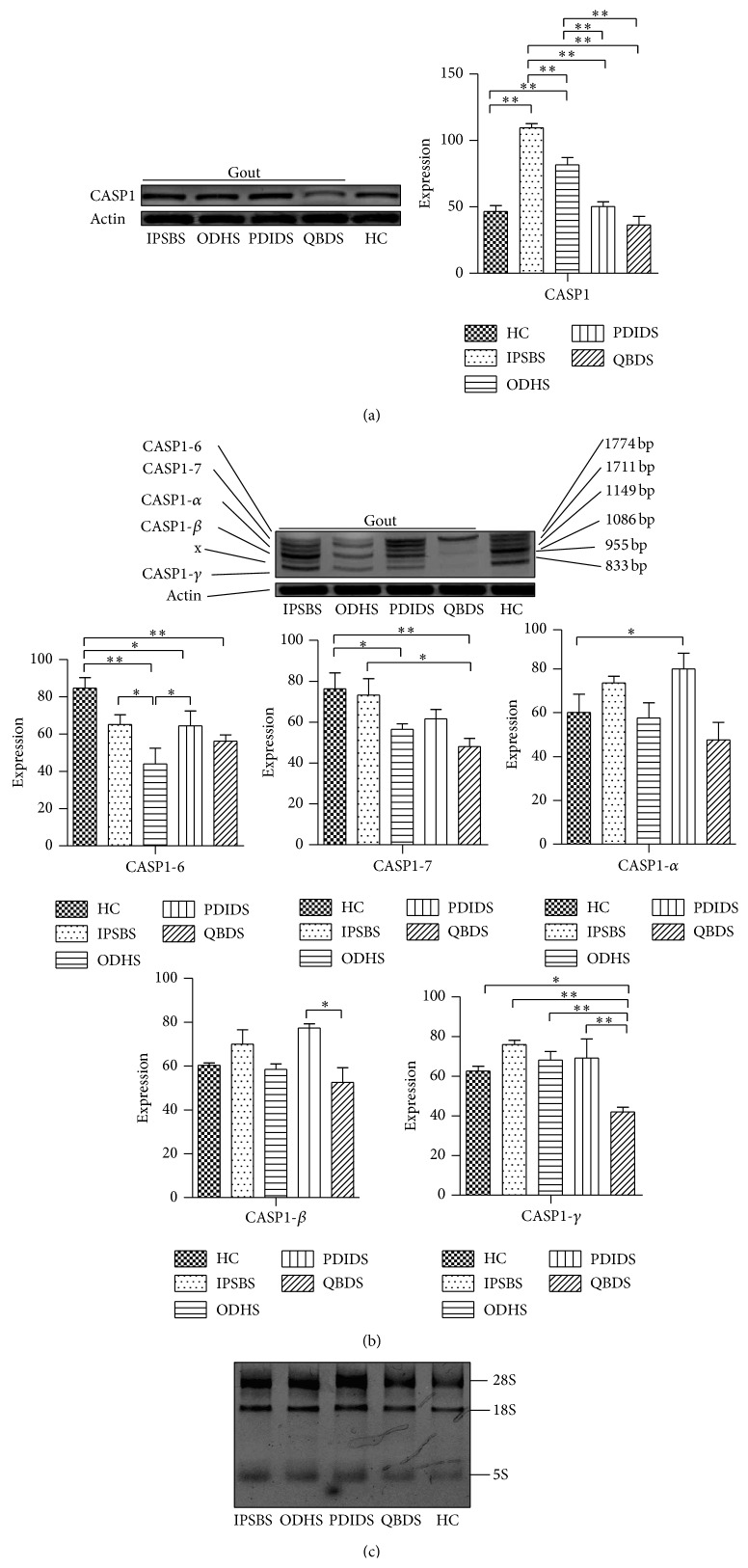
The mRNA expression of CASP1 gene and its transcript variant in PBMCs of PG patients with different TCM syndromes. Notes: ^∗^ *P* < 0.05; ^∗∗^ *P* < 0.01.

2.5 mL peripheral blood was taken and anticoagulated with heparin. PBMCs were separated by human lymphocytes separation liquid under sterile condition. According to instruction strictly, total RNA was extracted with Trizol reagent and the RNA was dissolved with 30 *μ*L no RNA enzymes water. 5 *μ*L RNA samples were taken and measured by agarose gel electrophoresis and three bands were showed in 1.5% agarose gel map: 28S, 18S, and 5S (Figures 2(c) and 3(c)). The absorbance value (*A* value) of RNA was detected by UV spectrophotometer detection at 260 and 280 nm wavelength and the *A*_260_/*A*_280_ ratio was calculated (adopted 1.8 to 2.0) [9]. According to instruction strictly, cDNA was synthesized with reverse transcription kits on condition of 37°C for 15 min and 85°C for 5 sec, and then the reaction was terminated. 60 *μ*L of the RT system was recorded as follows: 6 *μ*L 5 ×g DNA eraser buffer, 3 *μ*L gDNA eraser, 5 *μ*L total RNA, 12 *μ*L 5× PrimeScript buffer 2 (qRT-PCR), 3 *μ*L prime script RT enzyme mix I, 3 *μ*L RT primer mix, and 28 *μ*L RNase free dH_2_O [10]. The cDNA product was stored at −20°C.

### 2.6. Measurement of CASP1 Gene, Transcript Variant, and IL-1*β* by RT-PCR

PCR amplification was made in the 25 *μ*L reaction system, which was created according to cDNA of HC and patients with PG: 0.25 *μ*L TaKaRa LA Taq (5 U/*μ*L), 2.5 *μ*L 10× LA PCR buffer II (Mg^2+^ Free), 2.5 *μ*L MgCl_2_ (25 mM), 4 *μ*L dNTP mixture (each 2.5 mM), 1 *μ*L template DNA (cDNA), 0.5 *μ*L primer 1 (upstream 20 *μ*M), 0.5 *μ*L primer 2 (downstream 20 *μ*M), and 13.75 *μ*L sterilized and distilled water. The reaction condition was initial denaturation in 95°C for 5 min, 94°C for 30 sec, 55°C for 30 sec, and 72°C for 1 min and repeated 35 cycles, and extension in 72°C for 5 min [9]. Amplification products were measured by 1% agarose gel electrophoresis and agarose gel was shot with exposure by FUSION-Fx5 lithography machine. The gray value of the exposed PCR strip images was measured by BIO-RAD Quantity-One software. The ratio of gray value of target gene to internal parameters was to reveal the expression level of target gene and its transcript variant mRNA.

### 2.7. Measurement of CASP1 and IL-1*β* Gene by qRT-PCR

cDNA of HC and patients with PG was measured by qRT-PCR instrument to create 20 *μ*L reaction system: 10 *μ*L SYBR Premix Ex Taq II, 0.4 *μ*L ROX Reference Dye II, 0.8 *μ*L upstream primer (10 *μ*mol/L), 0.8 *μ*L downstream primer (10 *μ*mol/L), and 8 *μ*L sterilized and distilled water. The reaction condition was 95°C for 10 min, 95°C for 15 s, and 60°C for 1 min and repeated 40 cycles. Each specimen was done with multiple pores and the C_t_ value difference between the multiple pores was controlled within 0.5. All amplifications were performed on the ABI 7900 real-time PCR instrument. The melting curve was made after the amplification. ΔC_t_ derived from C_t_ value minus internal parameters of the target gene and 2^−ΔC_t_^ value represented the expression level of the target gene mRNA (the amplification of specific primer of target gene and its transcript variant).

### 2.8. The Purification and Sequencing of the PCR Products

The PCR products were purified and recycled by agarose gel purification. The recycled gene fragments were remeasured by agarose gel electrophoresis. After bands were confirmed, the nucleic acid sequence of purified target gene fragments was sequenced in Genscript Biotechnology Co. Ltd.

### 2.9. Measurement of the Level of Plasma IL-1*β* by ELISA Kit

According to instructions strictly, ELISA kit was operated, and OD values of each hole was measured with a microplate reader at 450 nm. The standard curve was made with standard sample of kit. The corresponding concentration was identified according to the absorbance value of the sample, and the final concentration of the sample was calculated by multiply the measured concentration by dilution factor.

### 2.10. Correlation Analysis

We analyzed the correlation between mRNA expression of CASP1 gene and its transcript variant and IL-1*β* in patients with PG in different phases and TCM syndromes and also analyzed the correlation between expression of CASP1 gene and its transcript variant mRNA and IL-1*β* protein in patients with PG in different phases and TCM syndromes.

### 2.11. Statistical Analysis

SPSS 16.0 software package was used for statistical analysis and all data were presented as mean ± standard deviation ($\overset{-}{x}\pm s$). Comparison of mean values among multiple groups was done with ANOVA and comparison between two groups was done with the LSD test. The correlation of each group was done with spearman analysis. A *P* < 0.05 was considered as significant difference among groups.

## 3. Results

### 3.1. The Comparison of the Results between Different Phases and TCM Syndromes of Patients with PG

See Table 2.

### 3.2. The Primers Amplified Results of CASP1 Gene and Its Transcript Variant in PBMCs of Patients with PG in Different Phases and TCM Syndromes

See Figures 2 and 3.

### 3.3. The Expression of CASP1 Gene and Its Transcript Variant mRNA in PBMCs of PG Patients with PG in Different Phases and TCM Syndromes

The expression of CASP1 mRNA in APPG group was significantly higher than that in HC group (*P* < 0.01), the expression of CASP1-6 mRNA in APPG group and CASP1-6 and CASP1-7 mRNA in NAPPG group was significantly lower than in HC group (*P* < 0.01 or *P* < 0.05); the expression of CASP1 and CASP1-gamma mRNA in NAPPG group was significantly lower than in APPG group (*P* < 0.01 or *P* < 0.05, Figure 2).

The expression of CASP1 gene mRNA in IPSBS and ODHS group was significantly higher than in HC group (*P* < 0.01); the expression of CASP1-6 and CASP1-7 mRNA in ODHS and QBDS group and CASP1-6 mRNA in PDIDS group all was significantly lower than in HC group (*P* < 0.05 or *P* < 0.01); the expression of CASP1-6 and CASP1-7 mRNA in ODHS group, CASP1 mRNA in PDIDS group, and CASP1, CASP1-7, and CASP1-gamma mRNA in QBDS group all was significantly lower than in IPSBS group (*P* < 0.05 or *P* < 0.01); the expression of CASP1 mRNA in PDIDS and CASP1-7 and CASP1-gamma mRNA in QBDS group all was significantly lower than in ODHS group (*P* < 0.05 or *P* < 0.01); the expression of CASP1-6 mRNA in PDIDS group was significantly higher than in ODHS group (*P* < 0.05); the expression of CASP1-beta and CASP1-gamma mRNA in QBDS group was significantly lower than in PDIDS group (*P* < 0.05 or *P* < 0.01, Figure 3).

### 3.4. The Expression of IL-1*β* mRNA in PBMCs of Patients with PG in Different Phases and TCM Syndromes

**Figure 4:**
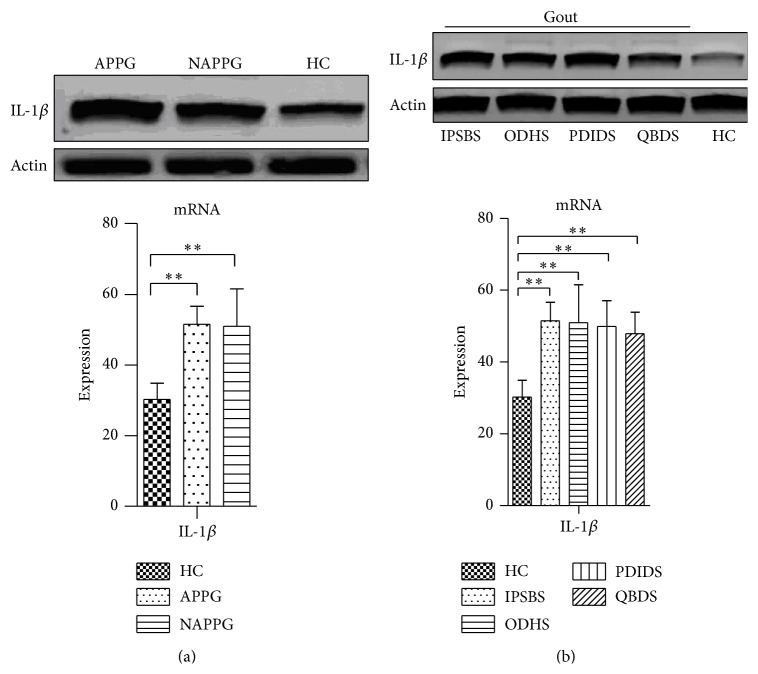
The mRNA expression of IL-1*β* in PBMCs of patients with PG in different phases and TCM syndromes. Notes: (a) and (b) IL-1*β* gene primers were amplified to one fragment (73 bp); (a) the mRNA expression of IL-1*β* in PBMCs of patients with PG in different phases; (b) The mRNA expression of IL-1*β* in PBMCs of patients with PG in different TCM syndromes.  ^∗∗^ *P* < 0.01.

The expression of IL-1*β* mRNA in APPG and NAPPG group was significantly higher than in HC group (*P* < 0.01, Figure 4(a)).

The expression of IL-1*β* mRNA in IPSBS, ODHS, PDIDS, and QBDS group was significantly higher than in HC group (*P* < 0.01, Figure 4(b)).

### 3.5. The Expression of Plasma IL-1*β* Protein of Patients with PG in Different Phases and TCM Syndromes

**Figure 5:**
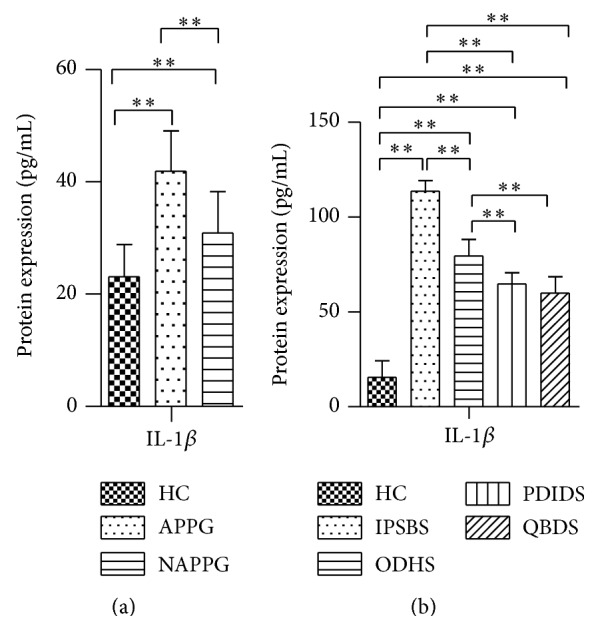
The expression of plasma IL-1*β* protein in patients with PG in different phases and TCM syndromes. Notes: (a) the expression of plasma IL-1*β* protein in patients with PG in different phases; (b) the expression of plasma IL-1*β* protein in patients with PG in different TCM syndromes.  ^∗∗^ *P* < 0.01.

The expression of plasma IL-1*β* protein in APPG and NAPPG group was significantly higher than in HC group (*P* < 0.01), and the expression of plasma IL-1*β* protein in APPG group was significantly higher than in NAPPG group (*P* < 0.01) (Figure 5(a)).

The expression of IL-1*β* protein in IPSBS, ODHS, PDIDS, and QBDS group was significantly higher than in HC group (*P* < 0.01); the expression of IL-1*β* protein in IPSBS group was significantly higher than in ODHS group (*P* < 0.01); the expression of IL-1*β* protein in IPSBS and ODHS group was significantly higher than in PDIDS and QBDS group (*P* < 0.01) (Figure 5(b)).

### 3.6. The Results of Correlation Analysis

**Figure 6:**
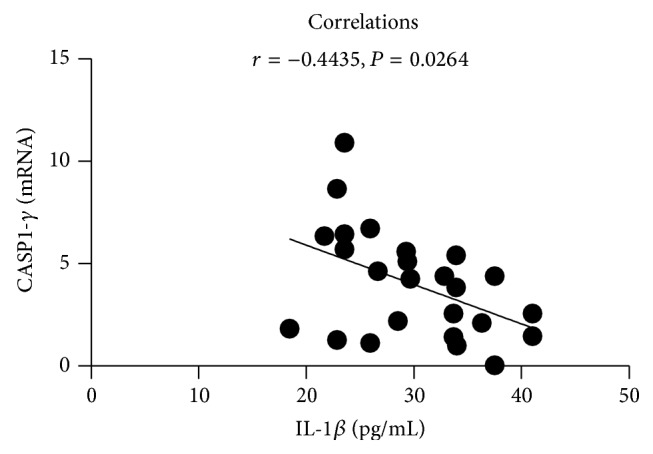
Correlation analysis of the expression level between CASP1-gamma gene transcript variant mRNA and IL-1*β* protein in APPG group.

Correlation analysis showed that there was negative correlation between the expression of CASP1-gamma gene transcript variant mRNA and IL-1*β* protein in APPG group (*r* = −0.4435, *P* = 0.0264; Figure 6), and no significant correlation was observed between the mRNA expression of CASP1 gene and its transcript variant and IL-1*β* in other groups (*P* > 0.05).

## 4. Discussion

Gout has a strong influence on people's health. The primary gout, with certain familial predisposition, was caused by both genetic and environmental factors, and the etiology was unknown except for about 1% due to congenital defects in purine metabolism enzymes [14]. In recent years, the incidence of gout in adults in China increases year by year and also increases with age [14]. Chinese medicine believes that the causes of gout were congenital deficiency, being worn out with age, dysfunction of spleen in transportation and with no ability to ascend lucidity or descend turbidity, or deficiency of kidney for activation of Qi and not distinguishing lucidity and turbidity resulted in cereal essence not being reformed along with noxious dampness which stranded and accumulated in the body, related to the dysfunction of middle jiao and lower jiao. By classifying gout patients into Western medicine phases and TCM syndrome, we found that the main TCM syndrome in the acute and nonacute phase of gout patients was IPSBS, ODHS, PDIDS, and QBDS, among which ODHS and IPSBS were mainly in the acute phase of gout while PDIDS and QBDS were in the nonacute phase. The results showed that the main syndromes in the acute phase of gout were obstruction of dampness and heat syndrome and intermingled phlegm-stasis blood syndrome, while, in the nonacute phase of gout, the main syndromes were Pi-deficiency induced dampness syndrome and Qi-blood deficiency syndrome.

Some researches had shown that inflammation and immunity played a certain role in the pathogenesis of gout [2], and the IL-1*β* level in peripheral venous blood of gout patients had significantly increased [15]. The body's innate immunity uses Toll-like Receptors (TLRs) to recognize the “naked” MSU crystals which can activate myeloid differentiation factor (MyD88) dependent NF-*κ*B pathway and lead the gene transcription to produce prointerleukin 1*β* (Pro-IL-1*β*) precursor. Pro-IL-1*β* is cut into mature IL-1*β* through CASP1. By binding to IL-1 receptor, IL-1*β* can activate the IL-1 and NF-*κ*B signal pathway, which can cause a large expression of proinflammatory factor like IL-1*β*, tumor necrosis factor-*α* (TNF-*α*), and so on, producing inflammatory cascade amplification effect [16].

It was known that an mRNA precursor (pre-mRNA) could produce different mRNA splice variant by selecting different splicing sites, and different splice variant plays an important role in the occurrence and development of diseases [17–19]. Studies showed that the immature CASP1 mRNA could be translated into six different subtypes through variable shear and transcription: alpha, beta, gamma, delta, epsilon, and zeta, all of which can not only mediate inflammatory response but also play different roles in cell death [20, 21]. Our study retrieved the already known seven gene transcript variants of CASP1 gene from Genbank. After CASP1 gene and its transcript variant primers were designed and measured by RT-PCR, we found that the expression of CASP1 gene mRNA in IPSBS and ODHS group was significantly higher than in HC group (*P* < 0.01); the expressions of CASP1-6 and CASP1-7 mRNA in ODHS group, CASP1-6 mRNA in PDIDS group, and CASP1-6 and CASP1-7 mRNA in QBDS group were all significantly lower than in HC group (*P* < 0.05 or *P* < 0.01); the expression of CASP1 mRNA in APPG group was significantly higher than in HC group (*P* < 0.01), the expression of CASP1-6 mRNA in APPG group and CASP1-6 and CASP1-7 mRNA of NAPPG group was significantly lower than in HC group (*P* < 0.05 or *P* < 0.01); in the meantime, the expression of IL-1*β* mRNA in patients with PG in different phases and TCM syndromes was significantly higher than in HC group (*P* < 0.01). These results showed that CASP1 gene and its transcript variant are expressed abnormally in patients with PG in different phases and TCM syndromes, and these results also suggested that CASP1 gene and its transcript variant might play an important role in the regulation of inflammatory responses in patients with PG. The study also found that there are differences of the expression of CASP1 gene and its transcript variant between APPG and NAPPG groups. The results showed that the change of phases and TCM syndromes of patients with PG may relate to the change of the expression of CASP1 gene and its transcript variant.

Our study also found that the protein expression of plasma IL-1*β* of patients with PG in different phases and TCM syndromes was significantly higher than in HC group (*P* < 0.01); there were differences between the expression of plasma IL-1*β* protein in patients with PG in different phases and TCM syndromes, and the correlation analysis showed that there was negative correlation between the expression of CASP1-gamma gene transcript variant mRNA and IL-1*β* protein in APPG group (*r* = −0.4435; *P* = 0.0264). The results suggested indirectly that the CASP1 gene transcript variant may play an important role in the inflammatory responses of patients with PG, and the mechanism needs further and in-depth study [22, 23].

In conclusion, obstruction of dampness and heat syndrome and intermingled phlegm-stasis blood syndrome tend to occur in the acute phase of gout, while Pi-deficiency induced dampness syndrome and Qi-blood deficiency syndrome tend to occur in the nonacute phase of gout, and the mechanism may relate to the dysregulated expression of CASP1 gene and its transcript variant; the expression change of CASP1 gene and its transcript variant may be associated with the onset of gout. Therefore, further study for the mechanism of CASP1 gene transcript variant in PG is expected to provide new method for the effective prevention and treatment of PG.

## 5. Limitations

A small sample size may be a limitation for the present study. CASP1 transcript variant primers are not specific and cannot be detected by qRT-PCR; there may have been an error in the results detected by RT-PCR. Hence, every transcript variant specific primer of CASP1 should be redesigned later, and CASP1 gene transcript variant should be detected by qRT-PCR.

## 6. Conclusions

In summary, through our research, we initially demonstrated the existence of CASP1-6, CASP1-7, CASP1-alpha, CASP1-beta, and CASP1-gamma transcript variant in PBMCs of gout patients, and the expression of each transcript variant mRNA showed difference between patients with gout in different TCM syndromes and health controls, combined with relevant laboratory index; the results preliminarily indicated that CASP1 gene and its transcript variant might play an important regulating role in the pathogenesis of gout.

## Figures and Tables

**Table 1:** Primer sequences of CASP1 gene and its transcript variant.

Gene (transcript variant) name	Upstream	Downstream	Genetic fragment size
CASP1	5′-CGCAGATGCCCACCACT-3′	5′-TGCCCACAGACATTCATACAG-3′	96 bp
CASP1-6 (NM_001257118.2)	5′-TACAGTTATGGATAAGACCCGAGC-3′	5′-GCAGACATAATTCCAAAAACCTTTA-3′	1774 bp
CASP1-7 (NM_001257119.2)	5′-TACAGTTATGGATAAGACCCGAGC-3′	5′-GCAGACATAATTCCAAAAACCTTTA-3′	1711 bp
CASP1-alpha (NM_033292.3)	5′-TACAGTTATGGATAAGACCCGAGC-3′	5′-GCAGACATAATTCCAAAAACCTTTA-3′	1149 bp
CASP1-beta (NM_001223.4)	5′-TACAGTTATGGATAAGACCCGAGC-3′	5′-GCAGACATAATTCCAAAAACCTTTA-3′	1086 bp
CASP1-gamma (NM_033293.3)	5′-TACAGTTATGGATAAGACCCGAGC-3′	5′-GCAGACATAATTCCAAAAACCTTTA-3′	833 bp
IL-1*β*	5′-ACAGATGAAGTGCTCCTTCCA-3′	5′-GTCGGAGATTCGTAGCTGGAT-3′	73 bp
*β*-actin	5′-GAGCTACGAGCTGCCTGACG-3′	5′-GTAGTTTCGTGGATGCCACAG-3′	120 bp

**Table 2:** The comparison of the results between the different phases and TCM syndromes of patients with PG.

Phases	APPG (*n* = 44)				NAPPG (*n* = 52)			
TCM syndromes	ODHS	IPSBS	PDIDS	QBDS	PDIDS	QBDS	IPSBS	ODHS
*n*	16	12	10	6	21	14	10	7
Percent (%)	36.36	27.27	22.73	13.64	40.38	26.92	19.23	13.46

Notes: APPG: acute phase primary gout; NAPPG: nonacute phase primary gout; ODHS: obstruction of dampness and heat syndrome; IPSBS: intermingled phlegm-stasis blood syndrome; PDIDS: Pi-deficiency induced dampness syndrome; QBDS: Qi-blood deficiency syndrome.

## Abbreviations

ACR: American Rheumatism Association
AP: Acute phase
APPG: Acute phase primary gout
CASP1: Caspase-1
DAMPs: Danger-associated molecular patterns
HC: Healthy control
IPSBS: Intermingled phlegm-stasis blood syndrome
LPS: Lipopolysaccharides
LSD: Least significant difference
MSU: Monosodium urate
NAP: Nonacute phase
NAPPG: Non-acute phase primary gout
ODHS: Obstruction of dampness and heat syndrome
PBMCs: Peripheral blood mononuclear cells
PDIDS: Pi-deficiency induced dampness syndrome
PG: Primary gout
Pro-IL-1*β*: Prointerleukin 1*β*
QBDS: Qi-blood deficiency syndrome
TCM: Traditional Chinese medicine
TLRs: Toll-like receptor-specific.
